# Editorial: Multiparametric stress echocardiography in stable CAD patients. How technological advances may transform diagnosis and prognosis

**DOI:** 10.3389/fcvm.2026.1811863

**Published:** 2026-04-10

**Authors:** Nicola Gaibazzi, Attila Kardos

**Affiliations:** 1 Cardiology Department, Parma University Hospital, Parma, Italy; 2 Department of Cardiology, Translational Cardiovascular Research Group, Milton Keynes University Hospital NHS Foundation Trust, Milton Keynes, United Kingdom; 3 Faculty of Medicine and Health Sciences, University of Buckingham, Buckingham, United Kingdom

**Keywords:** artificial intelligence, CFVR, global longitudinal strain (GLS), multi-parametric stress echocardiography, myocardial contrast echocardiography

## Introduction

Precise evaluation of myocardial ischemia—encompassing both regional and global myocardial functional assessment—is fundamental in the diagnostic workup and management of patients with suspected or established stable coronary artery disease (CAD). This comprehensive approach is crucial for accurate diagnosis, risk stratification, and the formulation of evidence-based management strategies. Conventional echocardiographic techniques, which primarily rely on visual assessment of wall motion and thickening, provide significant specificity for severe, flow-limiting coronary stenoses but demonstrate limited sensitivity for less severe disease. As such, advanced imaging modalities capable of integrating multiple physiological and anatomical parameters are increasingly sought to overcome these limitations and refine clinical decision-making.

Multiparametric echocardiography (MPE) represents a novel, radiation-free, and cost-efficient imaging technique that synthesizes a spectrum of echocardiographic markers—including myocardial deformation, coronary flow velocity reserve, contrast myocardial perfusion, and advanced hemodynamic measurements—into a unified diagnostic platform. By leveraging these diverse parameters, MPE offers the potential for enhanced diagnostic precision and improved prognostic stratification compared to conventional echocardiography. While preliminary research supports its promise, rigorous validation in routine clinical practice remains necessary to establish its definitive utility for the diagnosis and management of CAD.

## Defining multiparametric echocardiography

Multiparametric echocardiography (MPE) refers to an advanced echocardiographic approach that integrates multiple ultrasound-based parameters to enhance the diagnostic and prognostic evaluation of cardiac function, particularly in patients with suspected or established coronary artery disease (CAD). Unlike conventional echocardiography, which relies predominantly on visual assessment of regional and global wall motion, MPE synthesizes a range of quantitative and qualitative markers to provide a more comprehensive evaluation of myocardial structure, function, perfusion, and hemodynamics.

Standard rest and stress echocardiography primarily detect wall motion abnormalities either at baseline or during provocation (exercise or pharmacologic stress), which is highly specific for severe, flow-limiting CAD but lacks sensitivity for milder degrees of coronary stenosis. Multiparametric stress echocardiography addresses these limitations by incorporating additional functional and physiological variables, thereby increasing diagnostic accuracy and expanding clinical utility (1).

The principal parameters assessed in multiparametric echocardiography include:

- Myocardial deformation imaging—primarily strain analysis via speckle-tracking to quantify regional and global myocardial function.
- Doppler Coronary Flow Velocity Reserve (CFVR)—measurement of coronary microvascular function and flow reserve.
- Contrast myocardial perfusion imaging—qualitative and quantitative assessment of myocardial blood flow using ultrasound contrast agents.
- Comprehensive cardiac hemodynamics—advanced Doppler and strain-derived parameters for detailed evaluation of filling pressures, volumes, and myocardial work.
- Automated AI-based wall motion analysis—application of artificial intelligence algorithms to improve objectivity and reproducibility in the assessment of wall motion and volumetric measurements.

The central hypothesis underlying multiparametric echocardiography is that the integration of diverse echocardiographic biomarkers—or “echo-omics”—may improve diagnostic yield, facilitate risk stratification, and ultimately inform and optimize the management of patients with CAD. However, large-scale randomized clinical trials are required to determine the true clinical value of this approach.

*The Role of Post-Systolic Strain and ECG Changes During Dobutamine Stress Echocardiography: Editorial Perspective - Insights from a Negative Study on Diagnostic Accuracy for Symptomatic Coronary Artery Disease*.

In their recent investigation, Zivanic et al. examined whether the post-systolic strain index, an automatically derived parameter from speckle-tracking echocardiography software, could enhance the diagnostic performance of dobutamine stress echocardiography (DSE) for detecting symptomatic coronary artery disease (CAD). This study stands out for its scientific rigor and transparency, as it reports a “negative” result—demonstrating that post-systolic strain index does not improve the overall accuracy of DSE in diagnosing CAD among patients presenting with angina.

The findings are particularly relevant in the context of multiparametric echocardiography, where integration of novel quantitative markers is often expected to advance diagnostic precision. However, the present study underscores that not all emerging indices translate into clinical utility. Zivanic et al. show that despite the technological sophistication of post-systolic strain measurement, its addition to stress echocardiographic protocols does not yield incremental value for routine CAD detection. This is a critical contribution, as it helps focus time-constrained cardiology practice on parameters and methods with proven impact on patient management and outcomes.

Importantly, the publication of such negative results addresses the “positive study publishing bias” prevalent in cardiovascular research, reminding clinicians and researchers that the absence of incremental benefit is itself a meaningful finding. By highlighting which advanced measures are not worth pursuing in daily practice, this study supports evidence-based refinement of diagnostic pathways in stress echocardiography.

Future research should continue to rigorously test new indices, reporting both positive and negative outcomes, to ensure that advances in echocardiography translate into tangible benefits for patients.

*“Short-Term Outcomes of Non-ST Segment Elevation Acute Coronary Syndrome After Percutaneous Coronary Intervention: A Single-Center Speckle Tracking Echocardiographic Study in Vietnam”*.

In their single-center study, Trinh et al. investigated the short-term outcomes in patients with non-ST segment elevation acute coronary syndrome (NSTE-ACS) following percutaneous coronary intervention (PCI), utilizing speckle tracking echocardiography to assess global longitudinal strain (GLS). The main finding is that GLS, an easily obtainable and often automated measure of left ventricular longitudinal deformation, possesses prognostic significance for post-infarction patients with NSTE-ACS. This supports the role of GLS as an advanced echocardiographic parameter in risk stratification.

In the manuscript entitled “*The Impact of Type 2 Diabetes on Left Ventricular Function in Hypertensive Patients: A Three-Dimensional Speckle-Tracking Imaging Study*” Qingmei et al. provide important insights into the additive adverse effects of type 2 diabetes mellitus (T2DM) on left ventricular (LV) function in hypertensive individuals. Utilizing advanced three-dimensional speckle-tracking echocardiography (3D-STE), the authors demonstrate that T2DM significantly worsens both global and regional LV strain parameters in patients already burdened by hypertension. These findings are clinically relevant, as they highlight the capacity of 3D-STE to detect subtle, asymptomatic myocardial dysfunction at a preclinical stage—well before overt symptoms or conventional echocardiographic changes emerge.

The study reinforces the potential of strain imaging as a surrogate endpoint in cardiovascular research, particularly for populations at high risk of heart failure. However, despite its promise, the current clinical application of 3D strain imaging remains limited. A key barrier to broader adoption is the lack of inter-vendor standardization in the acquisition and analysis of 3D strain data. Addressing this gap through consensus protocols and technical harmonization will be essential to be able to use 3D-STE in the routine clinical practice.

In the paper entitled “*Exercise intolerance in patients with chronic coronary syndrome: Insights from exercise stress echocardiography”*, Zhu et al. conducted a comprehensive evaluation of exercise intolerance among patients with chronic coronary syndrome (CCS) using exercise stress echocardiography. The investigators systematically assessed a range of standard and advanced echocardiographic parameters, including conventional Doppler indices, tissue Doppler flow, myocardial strain, and myocardial work. Their analysis aimed to identify independent echocardiographic predictors of impaired exercise capacity in this population.

The principal finding was that, among the broad array of “echo-omics” explored, only peak end-diastolic volume index/E/e’ (EDVi/E/e’) and the change in stroke volume index (*Δ*SVi) during exercise emerged as independent predictors of exercise intolerance in CCS patients. These results suggest that the integration of these specific parameters into clinical practice could enhance risk stratification and tailor management strategies for such patients.

This hypothesis-generating study provides important guidance for future research, highlighting the potential for targeted selection of advanced echocardiographic measures in the evaluation of exercise capacity. Further validation in larger cohorts will be necessary to confirm the clinical utility of these findings and to refine the role of “echo-omics” in the management of CCS.

In the submitted study, “*Comparative Study of Myocardial Perfusion and Coronary Flow Velocity Reserve Derived from Adenosine Triphosphate Stress Myocardial Contrast Echocardiography in Coronary Lesions with No/Mild Stenosis*,” Liu et al. tackle a nuanced aspect of noninvasive coronary assessment by deploying adenosine triphosphate (ATP) as a vasodilator during myocardial contrast stress echocardiography—a stressor infrequently used in routine practice. Their research focuses on patients presenting with chest pain and angiographically normal or mildly stenotic coronary arteries, aiming to elucidate the relationship between qualitative myocardial perfusion and Doppler-derived coronary flow velocity reserve (CFVR) in the left anterior descending (LAD) artery.

Despite the study's modest sample size and the inherent subjectivity of visual perfusion assessments—susceptible to artifacts—the authors provide hypothesis generating observations. Notably, they report a lack of concordance between qualitative perfusion defects and reduced CFVR in the LAD territory. Most patients exhibiting perfusion abnormalities were found to have normal CFVR, highlighting a significant physiological mismatch. This divergence between myocardial perfusion and CFVR raises important questions about the underlying mechanisms and clinical implications for patients with angina with no occlusive CAD.

Overall, this investigation challenges prevailing assumptions about the correlation between microvascular perfusion and flow reserve, inviting further research to clarify the pathophysiological basis of such mismatch. The study underscores the need for more objective and standardized perfusion assessment modalities and calls for larger, multicenter trials to validate these findings.

In the study entitled “*Concordance of left ventricular volumes and function measurements between two human readers, a fully automated AI algorithm, and the 3D heart model*,” Myhre et al. rigorously evaluated the accuracy and reproducibility of echocardiographic assessments of left ventricular (LV) volumes and global longitudinal strain (GLS). The investigators compared measurements obtained via a commercially available automated AI software with those from expert human readers and a reference 3D heart model.

Their results demonstrated that AI-based measurements exhibited significantly lower inter-operator variability compared to human analysis. This finding underscores the potential of AI-assisted echocardiography to enhance the consistency and reliability of LV volumetric and functional parameters in clinical and research settings. Importantly, the authors highlight that AI-augmented echo readings may be particularly advantageous for retrospective studies, offering a streamlined and objective approach for comprehensive re-evaluation of archived imaging data.

These findings can signal a paradigm shift in cardiac imaging workflows. The adoption of AI-driven analysis tools could support higher diagnostic confidence, minimize observer-dependent discrepancies, and facilitate large-scale data analyses in contemporary echocardiography research and practice.

## Future perspectives

The integration of MPE into routine clinical practice for patients with known or suspected coronary artery disease demands rigorous standardization and validation through large-scale prospective randomized trials and comprehensive registries. Such initiatives will be pivotal in establishing the clinical value of MPE for diagnosis and risk stratification. Active collaboration between industry, researchers, and clinicians is crucial in developing affordable, efficient, bedside diagnostic tools that can support personalized risk assessment and guide therapeutic decisions. Ultimately, only those novel methods and parameters that withstand the scrutiny of robust clinical research will shape the future landscape of multiparametric echocardiography—ushering in an era of “echo-omics” characterized by precision and individualized care in cardiology.
